# Evaluating the Prevalence of Burnout Among Health Care Professionals Related to Electronic Health Record Use: Systematic Review and Meta-Analysis

**DOI:** 10.2196/54811

**Published:** 2024-06-12

**Authors:** Yuxuan Wu, Mingyue Wu, Changyu Wang, Jie Lin, Jialin Liu, Siru Liu

**Affiliations:** 1 Department of Medical Informatics West China Hospital Sichuan University Chengdu China; 2 Information Center West China Hospital Sichuan University Chengdu China; 3 West China College of Stomatology Sichuan University Chengdu China; 4 Department of Oral Implantology West China Hospital of Stomatology Sichuan University Chengdu China; 5 Department of Biomedical Informatics Vanderbilt University Medical Center Nashville, TN United States

**Keywords:** clinical decision support system, electronic health record, electronic medical record, health information technology, alert fatigue, burnout, health care professionals, health care service, EHR, systematic review, meta-analysis, health information system, clinician burnout, health informatics

## Abstract

**Background:**

Burnout among health care professionals is a significant concern, with detrimental effects on health care service quality and patient outcomes. The use of the electronic health record (EHR) system has been identified as a significant contributor to burnout among health care professionals.

**Objective:**

This systematic review and meta-analysis aims to assess the prevalence of burnout among health care professionals associated with the use of the EHR system, thereby providing evidence to improve health information systems and develop strategies to measure and mitigate burnout.

**Methods:**

We conducted a comprehensive search of the PubMed, Embase, and Web of Science databases for English-language peer-reviewed articles published between January 1, 2009, and December 31, 2022. Two independent reviewers applied inclusion and exclusion criteria, and study quality was assessed using the Joanna Briggs Institute checklist and the Newcastle-Ottawa Scale. Meta-analyses were performed using R (version 4.1.3; R Foundation for Statistical Computing), with EndNote X7 (Clarivate) for reference management.

**Results:**

The review included 32 cross-sectional studies and 5 case-control studies with a total of 66,556 participants, mainly physicians and registered nurses. The pooled prevalence of burnout among health care professionals in cross-sectional studies was 40.4% (95% CI 37.5%-43.2%). Case-control studies indicated a higher likelihood of burnout among health care professionals who spent more time on EHR-related tasks outside work (odds ratio 2.43, 95% CI 2.31-2.57).

**Conclusions:**

The findings highlight the association between the increased use of the EHR system and burnout among health care professionals. Potential solutions include optimizing EHR systems, implementing automated dictation or note-taking, employing scribes to reduce documentation burden, and leveraging artificial intelligence to enhance EHR system efficiency and reduce the risk of burnout.

**Trial Registration:**

PROSPERO International Prospective Register of Systematic Reviews CRD42021281173; https://www.crd.york.ac.uk/prospero/display_record.php?ID=CRD42021281173

## Introduction

The integration of electronic health record (EHR) into health care systems marks the beginning of a new era in medical information management, with significant potential benefits for patient care, clinical decision-making, and administrative efficiency [1,2]. EHR systems are central to the modern health care infrastructure [3]. Along with these benefits, however, the widespread adoption of EHR systems has raised concerns about the well-being of health care professionals [4,5]. Unintended consequences, such as burnout among health care professionals, technology-related errors, and increased safety risks, have been associated with EHR use [4,6,7]. In addition, a notable part of the problems with EHR systems in the United States is the need to provide additional documentation for insurance companies [8].

Within the realm of EHR use, burnout among health care professionals, characterized by emotional exhaustion, depersonalization, and a diminished sense of personal accomplishment, has emerged as a critical concern [9,10]. Burnout among health care professionals has become a pressing public health concern [11-13]. Some studies have reported an average burnout prevalence of 44% [2], with rates exceeding 80% in some specific settings and departments [4,5] such as primary care and emergency departments. This pervasive problem affects not only health care professionals but also patients, with negative consequences such as reduced quality of care and increased medical errors and psychological problems [14-17]. The estimated annual cost of burnout among health care professionals due to medical negligence and staff turnover exceeds US $4 billion [18].

The phenomenon of burnout among health care professionals goes beyond individual distress and has significant implications for patient safety, quality of care, and overall health system performance [14,15,19]. Understanding the prevalence and underlying factors of EHR-related burnout among health care professionals is critical to developing effective interventions and policy adaptations. These interventions are essential to mitigate this burden and ensure the long-term sustainability of EHR implementation in health care [19,20]. The increase in EHR-related burnout among health care professionals reflects a multifaceted interplay of factors, including increased documentation requirements, cumbersome user interfaces, and the rapid pace of technological development [9,16,18].

This systematic review and meta-analysis aims to provide a comprehensive assessment of the existing literature on EHR-related provider burnout. It seeks to capture the full extent of burnout, identify its causes, and provide evidence-based support and recommendations to alleviate this pervasive problem. In addition, we hypothesize that specific features of EHR systems, such as user interface design or increased documentation requirements, may contribute to provider burnout. We hope that this work will serve as a guide for health care organizations, policy makers, and EHR developers in developing interventions and technological improvements that prioritize the well-being of health care professionals. In doing so, we can promote a sustainable and resilient health care system while harnessing the potential benefits of EHR systems to improve patient care.

## Methods

### Study Guidelines

We focused on studies that directly measured burnout, as it is often considered in existing research to be a distinct emotional state, separate from depression or anxiety. This systematic review followed the PRISMA (Preferred Reporting Items for Systematic Reviews and Meta-Analysis) guidelines [21] and was registered with PROSPERO (CRD42021281173). Details of the guidelines and registration can be found in Multimedia Appendices 1 and 2, respectively.

### Definitions

Our definitions of burnout were based on the Maslach Burnout Inventory-Human Services Survey instrument (MBI-HSS) [13,22], which characterizes burnout with high emotional exhaustion as a score ≥27, high depersonalization as >10, and low personal accomplishment as <33. Across the included studies, burnout was defined inconsistently, with definitions ranging from any one of the 3 items to all 3 items. In cases where the same study examined multiple definitions of burnout, we used the most common definition (high emotional exhaustion, high depersonalization, and low personal accomplishment) for the meta-analysis. For alternative definitions, such as those from the Stanford Physician Wellness Survey [23] or mini-Z [24], only outcomes explicitly described as burnout were documented. We categorized studies according to the measurement tool and definition of burnout.

### Search Strategy

We systematically searched PubMed, Embase, and Web of Science to identify relevant peer-reviewed English language studies published between January 1, 2009, and December 31, 2022. We used several search terms to capture EHR systems, including “electronic health record” and its abbreviation “EHR,” as well as “electronic medical record (EMR)” and “computerized physician order entry (CPOE).” To capture the phenomenon of burnout, we used terms such as “burnout,” “alert fatigue,” and “exhaustion.” In defining our study participants, we considered a spectrum of health care professionals, including “doctor,” “clinician,” “physician,” “surgeon,” “medical staff,” and “health care provider.” On June 30, 2023, the researchers conducted a literature search in databases such as PubMed, Embase, and Web of Science, following the previously established search strategy. No papers were found that met the inclusion criteria for this review.

The terms were combined using Boolean logic and then filtered by publication date and language (English). A full description of the search strategy can be found in Multimedia Appendix 3. In addition, we carefully examined the references of each article and manually added 5 relevant references to our review list. Duplicate studies were systematically excluded from consideration.

### Inclusion and Exclusion Criteria

**Figure 1:**
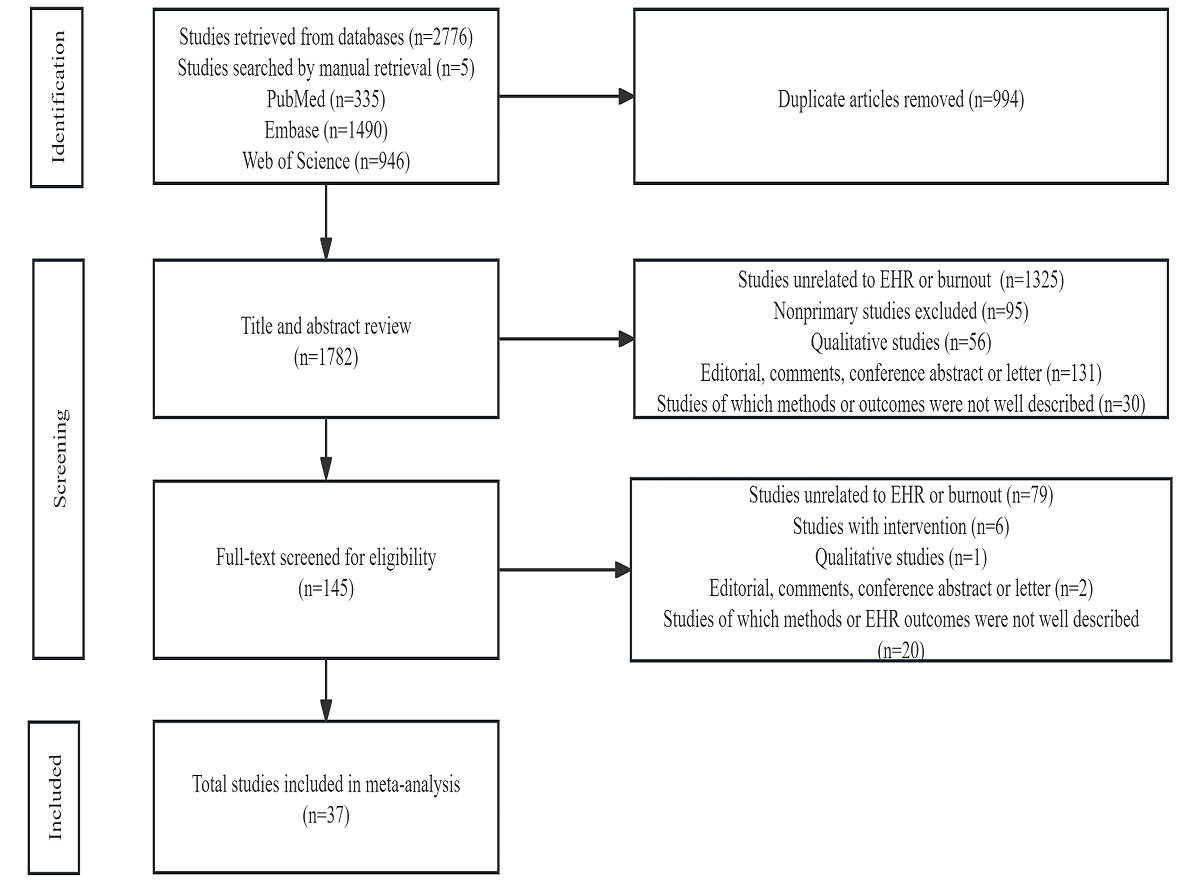
Flowchart of study selection. EHR: electronic health record.

Figure 1 shows the search and selection process. We applied strict inclusion and exclusion criteria to identify original and observational studies relevant to our research objectives. We included studies that examined general EHR use or specific supporting systems such as computerized physician order entry. We focused on studies that directly assessed burnout among health care professionals and individual psychological responses to EHR systems. Our review included the following types of research: cohort studies, case-control studies, and cross-sectional studies. EHR-related burnout was assessed using validated tools such as the MBI-HSS, the mini-Z, or other similar measures. The following publication types were excluded: abstracts, editorials, letters, reviews, commentaries, guidelines, and studies by non–health care professionals. In addition, studies were excluded if the necessary data could not be obtained from the corresponding author. We also excluded studies that repeated data already published in the literature.

Two reviewers independently screened all titles and abstracts to assess for relevance. Full texts of articles identified for further review were then assessed against the inclusion criteria. In cases of disagreement about the study eligibility of studies, a third reviewer was consulted for resolution.

### Data Extraction and Synthesis

For the included studies, we extracted relevant information including study design, geographical region, study duration, medical specialties involved, sample size, and relevant outcomes. The main outcomes included the prevalence of burnout in cross-sectional studies, the odds ratio (OR) along with its 95% CI in case-control studies, and the factors influencing burnout. We also documented the specific tools or measures used to assess these outcomes.

### Risk of Bias Assessment

Two reviewers assessed the integrity, confirmability, and quality of the cross-sectional studies using the Joanna Briggs Institute (JBI) checklist [25] and the Newcastle-Ottawa Scale (NOS) [26] for the included case-control studies. Details of these assessments can be found in Multimedia Appendices 4-6, respectively.

### Statistical Analysis

Meta-analysis was performed using R software (version 4.1.3; R Foundation for Statistical Computing). Heterogeneity was calculated using the Cochran *Q* test, and statistical significance was set at *P*<.05. If there was no statistical heterogeneity (*I*^2^<50%), the fixed-effects model was used to pool results; otherwise (*I*^2^>50%) the random-effects model was used [27,28]. We grouped the main outcomes according to the predictor and moderator factors described by the participants and derived from the outcome reports. Continuous variables were summarized using the mean and standardized mean difference, whereas rates were extracted for categorical variables. For cross-sectional studies, the effect size measure was the prevalence of burnout and its corresponding 95% CI. For case-control studies, the effect of EHR was assessed using the pooled OR and its 95% CI. Publication bias was analyzed using the Egger test [29] and the trim-fill funnel plot. A sensitivity analysis was performed for each omitted method to determine the robustness and reliability of the results.

## Results

### Characteristics of the Included Studies

After reviewing a total of 2776 studies, 37 were selected for inclusion in our analysis (Figure 1) according to the predefined criteria and after elimination of duplicates. The baseline characteristics of the selected studies are summarized in Tables 1 and 2. For further details see Multimedia Appendix 7 [6,30-60].

The studies included in our review covered the period from 2009 to 2022 and included regions in both Canada and the United States. They involved a total of 66,556 health care professionals. The sample sizes of these studies varied widely from 84 to 25,018 participants, and the response rates ranged from 8.9% to 73.0%.

The primary measure used to assess burnout in the majority of studies was the MBI-HSS, which was used in 17 of 37 studies (46%). In addition, the mini-Z scale was used in 10 studies (27%). Notably, 2 studies using the MBI-HSS used cutoff definitions for burnout subcomponents that followed the standardized recommendations of the MBI-HSS.

**Table 1:** Characteristics of the cross-sectional studies.

Author	Data collection	Region	Participants	Sample (total)	Burnout cases	Burnout prevalence (%)
Tawfik et al [30]	2011	United States	Physicians and other clinician staff	6560	3586	54.66
Shanafelt et al [31]	2014	United States	Physicians	1934	517	26.73
Tawfik et al [32]	2015	United States	Physicians and other clinician staff	1165	624	53.56
Olson et al [33]	2016	United States	Physicians	282	127	45.04
Tai-Seale et al [34]	2016	United States	Physicians	107	41	38.32
Apaydin et al [35]	2016	United States	Physicians and other clinician staff	110	44	40
Livaudais et al [36]	2016	United States	Physicians and other clinician staff	557	267	47.94
Tran et al [37]	2017	United States	Physicians and other clinician staff	1792	465	25.95
Marckini et al [38]	2017	Canada and United States	Physicians	919	331	36.02
Gardner et al [39]	2017	United States	Physicians	208	51	24.52
Hilliard et al [40]	2017	United States	Physicians and other clinician staff	422	116	27.49
Higgins et al [41]	2017	United States	Residents	116	62	53.45
Czernik et al [42]	2017	United States	Residents	163	81	49.69
Hauer et al [43]	2018	United States	Physicians	122	44	36.07
Gajra et al [44]	2018	United States	Physicians	2468	539	21.84
Adler-Milstein et al [45]	2018	United States	Physicians	100	52	52
Somerson et al [46]	2018	United States	Residents	128	65	50.78
Melnick et al [47]	2018	United States	Physicians	203	78	38.42
Coleman et al [48]	2018	United States	Physicians	870	397	45.63
Abraham et al [49]	2018	United States	Nurses	368	134	36.41
Kondrich et al [50]	2018	Canada and United States	Physicians	872	360	41.28
Kroth et al [51]	2019	United States	Physicians and other clinician staff	856	276	32.24
Tajirian et al[6]	2019	Canada	Physicians and trainee	222	84	37.84
Mandeville et al [52]	2019	United States	Physicians and other clinician staff	396	100	25.25
Tiwari et al [53]	2019	United States	Physicians and other medical staff	15,505	5065	32.67
Sinha et al [54]	2019	United States	Physicians	103	41	39.81
Anderson et al [55]	2019	United States	Physicians and trainee	756	373	49.34
Nair et al [56]	2019	United States	Physicians	281	127	45.20
Jha et al [57]	2020	United States	Physicians and other medical staff	230	86	37.39
Esmaeilzadeh and Mirzaei [58]	2020	Iran	Physicians and other medical staff	416	206	49.52
Holzer et al [59]	2020	United States	Physicians and trainee	84	30	35.71
Wilkie et al [60]	2021	Canada	Physicians	457	106	23.19

**Table 2:** Characteristics of the case-control studies.

Author	Data collection	Participants	Region	Exposure	Sample (total)	Burnout cases	OR^a^ (95% CI)
Eschenroeder et al [61]	2020	Physicians	United States	After-hours EHR^b^ charting time per week >6 hours	25,018	7616	2.43 (2.30-2.57)
Sharp et al [62]	2019	Physician trainees	United States	Working hours per week >70 hours	502	159	2.80 (1.78-4.40)
Peccoralo et al [63]	2019	Clinical faculty	United States	Time spent on EHR outside work >90minutes	1346	385	1.90 (1.40-2.78)
Harris et al [64]	2017	Advanced practice registered nurses	United States	Insufficient time for EHR documentation	333	69	3.72 (1.78-7.80)
Robertson et al [65]	2015	Primary care workers	United States	Extra time spent on EHR per week >6 hours	585	216	2.90 (1.90-4.40)

^a^OR: odds ratio.

^b^EHR: electronic health record.

### Quality of Included Studies

The quality of the cross-sectional studies was assessed using the JBI checklist. Of the cross-sectional studies reviewed, only 16 had a response rate of more than 50%. In addition, 24 studies provided a clear and precise description of their inclusion and exclusion criteria for participants. Additionally, 32 cross-sectional studies provided a detailed and thorough statistical analysis of their data and results.

We used the NOS to assess the risk of bias and the overall quality of the case-control studies. In particular, one study failed to clarify its selection criteria for the control group and comparability with the exposed group, which resulted in a high risk of selection bias. Furthermore, none of the 5 case-control studies reported information on the nonresponse population, indicating a high risk of nonresponse bias. Overall, the risk of bias in the case-control studies was assessed as moderate. A full breakdown of the quality assessment for each study can be found in Multimedia Appendices 4 [6,30-60] and 5 [61-65].

**Figure 2:**
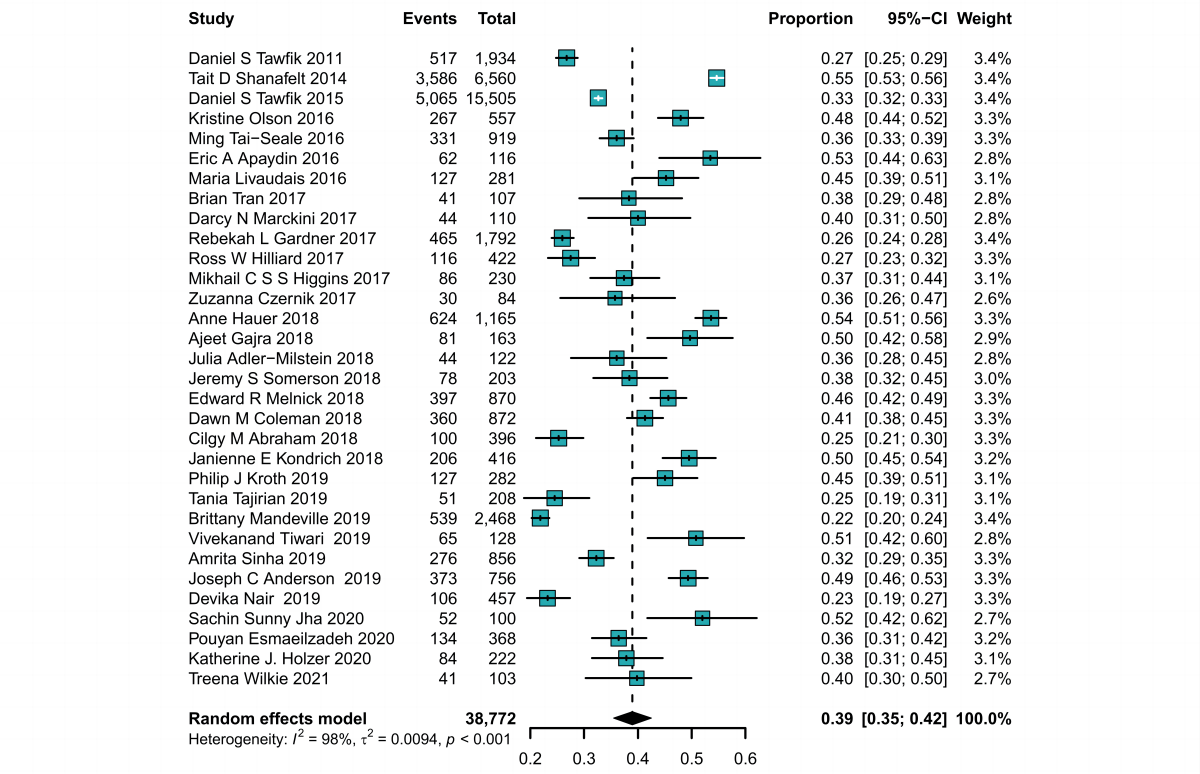
Forest plot of the pooled prevalence of burnout among health care professionals across cross-sectional studies [6,30-60]. IV: inverse variance methods.

**Figure 3:**
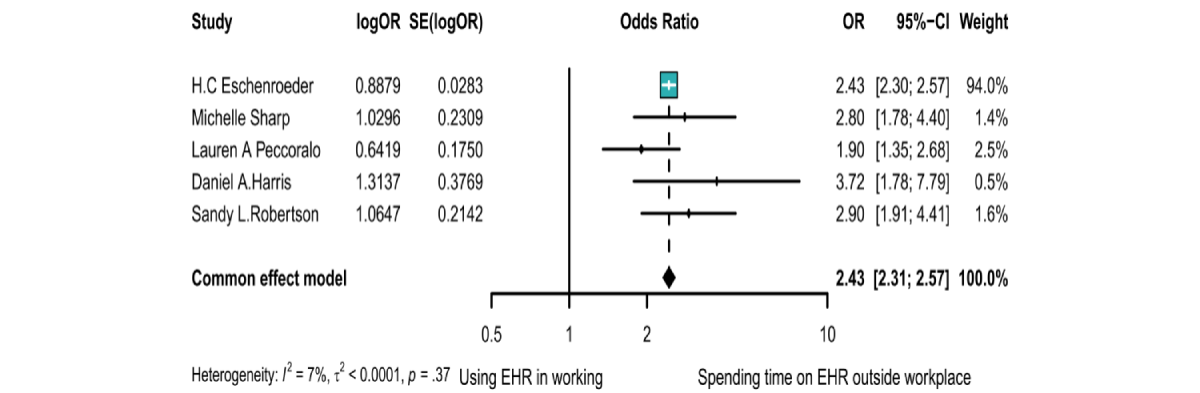
Measurement tool subgroup analysis of the pooled prevalence of burnout among health care professionals in cross-sectional studies [6,30-60]. IV: inverse variance methods; MBI: Maslach Burnout Inventory.

In our meta-analysis, we examined 37 studies that focused on identifying the prevalence of burnout associated with EHR use, involving a total of 66,556 health care professionals. The internal heterogeneity of 37 cross-sectional studies was evident in all included cross-sectional studies (*I*^2^=98.3%). Using random-effects models, we calculated the combined overall prevalence of EHR-related burnout of 40.4% (95% CI 37.6%-43.2%). Subgroup analysis showed that studies using the MBI-HSS reported a higher pooled prevalence of burnout (41.4%) than those using the mini-Z (35.1%) but lower than those using other instruments (43.2%). However, these differences were not statistically significant (Figures 2 and 3).

### Publication Bias

The Egger test and the funnel plot were used to estimate the publication bias in the included studies (*t*=1.35, *P*=.18), indicating no significant publication bias. The distribution of the points in the funnel plot is symmetric. There was no statistical difference in publication bias. The results are available in Multimedia Appendices 8 and 9.

### Sensitivity Analysis

Sensitivity analysis was performed using the individual omission method. The results showed that no single study had a significant effect on the pooled prevalence of burnout. The results of the sensitivity analysis indicated that the meta-analysis was robust.

### The Association Between Time Spent on the EHR and Burnout

**Figure 4:**
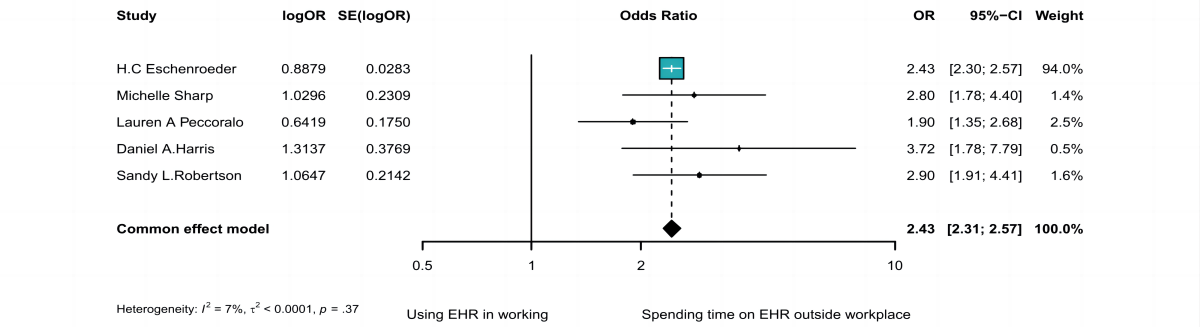
Forest plot of the association between the time spent using EHR and the risk of burnout [61-65]. EHR: electronic health record; IV: inverse variance methods; OR: odds ratio; SE: standard error of the TE; TE: take the logarithm of the effect value.

Data from 5 case-control studies with 27,784 participants were available for the meta-analysis of the time spent on EHR and burnout prevalence. There was no significant within-study heterogeneity (*I*^2^=7.2%, *P*=.37), and a longer duration of EHR use was associated with a higher prevalence of burnout (OR 2.43, 95% CI 2.31-2.57) (Figure 4).

### Main Causes of Burnout and Proposed Solutions

We have summarized the factors contributing to burnout among health care professionals in relation to EHR use in Table 3. Among these, challenges related to the design and availability of EHR systems were identified as the most significant contributors, as evidenced by 32 studies. Complaints from EHR users focused on several key issues: disruption to workflow [33]; cumbersome data entry (copy and paste) [59]; reduced direct communication with patients [38]; and annoyance with redundant, repetitive, or irrelevant alerts [52]. Poor EHR design has been shown to reduce work productivity and lead to prolonged EHR use [51,64]. This prolonged use has a negative impact on work-life balance of health care professionals and increasing burnout [42,57,65,66]. Workload factors, identified in 18 of the 32 studies, further exacerbate this problem. Specific aspects of workload that contribute to burnout include the number of hours worked per week [62-64], the frequency of night shifts [46,60], administrative documentation tasks [33,35,38,48,64], the volume of patient admissions [30,35,56], and the amount of information to manage in the EHR inbox [34,37,40]. Together, these factors exacerbate provider fatigue and increase the risk of burnout.

EHR usability, recognized as a contributing factor to burnout, relates to issues of accessibility and functionality of the system. This includes instances where the system is frequently unavailable due to maintenance, updates, or technical failures, as well as situations where the system is not user-friendly and requires excessive time to navigate and use effectively, potentially leading to burnout among health care professionals.

The factors contributing to burnout identified in the reviewed studies fall into 3 main categories: EHR use, work environment and organizational support, and the personal factors. Table 4 provides a summary of strategies to address these contributing factors. For example, the burden of medical clerical tasks imposed by EHR systems suggests the need to employ assistants or scribes to reduce the workload of health care professionals [31,67]. Evidence suggests that the EHR system itself can be improved by involving clinical staff in the design process [33], optimizing the user interface [39,64], minimizing the number of clicks required [52], and actively soliciting and incorporating user feedback [32]. In addition, some practitioners may not fully use health information technology in their roles and may be frustrated with EHR systems or similar systems [32]. To address this, health care organizations are advised to establish clear policies and procedures before implementing an EHR system and to provide ongoing health information technology education to reduce technology-related anxiety among users [32,52,68]. Finally, comprehensive and systematic initiatives are essential to effectively reduce burnout. Health care professionals are encouraged to work together to advocate for legislative and regulatory changes that ensure reasonable working hours, mandatory breaks, and safeguards against burnout [36,42,43,58,62].

Moreover, research also suggests that sociodemographic characteristics, interpersonal dynamics, and the work environment have a significant impact on the prevalence of burnout. In particular, factors such as being female, younger, and less experienced correlate with higher rates of burnout [34,48,55]. Conversely, high levels of satisfaction or positive perspectives on the use of EHR systems may reduce burnout [36,42,58].

**Table 3:** The influencing factors of burnout for studies.

Author	Design	Risk factors for burnout	Protective factors against burnout	Main EHR^a^ factors influencing burnout
Tawfik et al [30]	Cross-sectional	NICU^b^ with ≥10 weekly admissions, nursing care workload, and patient mortality	Burnout recognition education; implementation of burnout interventions at the individual and institutional level	Using EHR outside working or at home; time on using EHR
Shanafelt et al [31]	Cross-sectional	Using CPOE^c^, female gender, emergency medicine, each additional hour per week	Assistant order entry; documentation support	Time spent on clerical tasks
Tawfik et al [32]	Cross-sectional	HIT^d^ frustration, difficulty in falling asleep	Supplemental EHR training; scribes to assist documentation；team-based documentation and inbox management；automating data-entry tasks	Frustrated or stressed by EHR
Olson et al [33]	Cross-sectional	Poor control over workload, inefficient teamwork, lack of value alignment with leadership, and hectic-chaotic work atmosphere	Improve professional satisfaction; nonphysician order entry	Using EHR outside working or at home; insufficient documentation time
Tai-Seale et al [34]	Cross-sectional	Female gender and poor control over work schedule	Feeling highly valued; having good control over work schedule; working in a quiet or busy but reasonable environment; assist physician with email work; limit desktop medical work outside working hours (except in emergencies)	Using EHR outside working or at home; number of EHR system-generated in basket messages
Apaydin et al [35]	Cross-sectional	Managing unscheduled or same-day patients, lack of pharmacist support, administrative work, excessive overall workload, difficulty communicating with other professionals, inadequate care coordination, and answering patient emails	Interventions to facilitate provider-led quality improvement	Managing in-basket messages generated by EHR; responding to EHR alerts
Livaudais et al [36]	Cross-sectional	Negative perceptions of EHR	Perceiving positive effect of EHR in practice；technical support for EHR when using systems; EHR optimization program	Managing in-basket messages generated by EHR; poor EHR design; dealing with patient-call messages in systems
Tran et al [37]	Cross-sectional	Clinical full-time equivalents >0.9 and more incomplete messages in inbox	Perception positive attitudes about the effect of EHR or satisfied with EHR	Average additional 10 minutes spent on EHR after each visit; less efficient at completing EHR and inbox information
Marckini et al [38]	Cross-sectional	Female gender and dissatisfaction for clerical tasks	EHR optimization; improve physician efficiency; and job satisfaction	Managing in-basket messages generated by EHR; dissatisfaction with EHR
Gardner et al [39]	Cross-sectional	Primary care specialties, female gender, and reporting poor or marginal time for documentation	Perception positive attitudes about the effect of EHR or satisfied with EHR	Excessive data inputting in EHR; using EHR at home; frustrated with EHR
Hilliard et al [40]	Cross-sectional	High volume of patient call messages in the system and lack of control over workload	Copy and paste used in EHR documentation; assist with inbox tasks and create 2 administrative “desktops”	Using EHR outside working or at home; excessive data inputting in EHR; managing in-basket messages generated by EHR
Higgins et al [41]	Cross-sectional	Self-compassion, sleep disorder, lacking support from leaders, and poor control over schedules	Peer support, perceived appreciation and meaningfulness in work; maintaining values consistent with practice institution	Poor EHR usability; perception negative attitudes about the effect of EHR
Czernik et al [42]	Cross-sectional	Frustrated or stressed by EHR	Reducing the burden of documentation tasks; improving EHR usability; interventions to improve the EHR	Poor usability of EHR; information overload; degradation of medical documentation
Hauer et al [43]	Cross-sectional	Loss of practicing autonomy, female gender, frustrated with EHR, and increasing insurance and government regulation	Improve the functionality of EHR; enhance physician leadership and involvement; create a center for physician empowerment; create a physician health program	Using EHR outside workday
Gajra et al [44]	Cross-sectional	Variable reimbursement models, interactions with payers, and increasing treating and caring demands	Use advanced practice providers; hire additional administrative staff; invest in information technology	Excessive data inputting in EHR; frustrated or stressed by EHR; using EHR outside workday
Adler-Milstein et al [45]	Cross-sectional	Poor self-rated EHR skills	Improve EHR design; scribe or team documentation; reduce documentation requirements	Using EHR outside working or at home; time spent on EHR; system-generated in-basket messages (>114) per week
Somerson et al [46]	Cross-sectional	Working >80 hours per week, verbal abuse from faculty, educational debt, “scut” work >10 hours per week	Nursing support; duty-hour restrictions; improve EHR functionality and efficiency; adequate, personalized training and support; adequate social work support	Time spent on EHR per week; used EHR >20 hours per week
Melnick et al [47]	Cross-sectional	Practice location (academic medical center) and medical specialty	Improve EHR usability	Using EHR outside working or at home; poor EHR usability
Coleman et al [48]	Cross-sectional	Work-related physical pain, work-home conflict, and younger age	Build personal resilience, enhance wellness; peer support; reduce administrative or EHR burden	Using EHR outside working or at home; increased EHR or documentation requirement
Abraham et al [49]	Cross-sectional	Intraorganizational factors	EHR with multifunctional; reduce high EHR workload; work with supportive colleagues; improve team communication	High EHR workload
Kondrich et al [50]	Cross-sectional	Feeling undervalued by patients, lacking superior support, little promotion chances, perceived unfair clinical working schedule, and nonacademic environment	Improve physician well-being	Feeling that the EHR detracts from patient care
Kroth et al [51]	Cross-sectional	Overall stress	Improve EHR design; clinician training; scribes to assist documentation; work at home boundaries; exercise, taking breaks	Information overloading; slow system response; excessive data inputting；fail to navigate quickly; note bloat; patient- clinician relationship interference; fear of missing something; billing oriented notes.
Tajirian et al [6]	Cross-sectional	Workflow issues	Reduce the administrative burden of EHR；improve EHR	Lower satisfaction and higher frustration with the EHR；poor intuitiveness and usability of EHR
Mandeville et al [52]	Cross-sectional	HIT-related stress and burnout and emergency medicine	Improved workflow	Daily frustration added by EHR；using EHR outside working or at home
Tiwari et al [53]	Cross-sectional	Lack of physical exercise and weekly working hours	Teamwork and working satisfaction; self-care training	Poor EHR usability; dissatisfaction with EHR
Sinha et al [54]	Cross-sectional	Interpersonal disengagement	Lower CLOC^e^ ratio (total CLOC time to allocated appointment time); well-established personal resources	Using EHR outside working
Anderson et al [55]	Cross-sectional	Female gender, younger age, shorter practicing years, and having children at home	Taking 20 days or more of vacation time	Using EHR at home; ≥2-hour patient administration
Nair et al [56]	Cross-sectional	Working long hours, weekly number of nursing patients, practice environment, disinterested health systems, and dissatisfaction with remuneration	Caring for fewer patients per week	Using EHR outside working or at home; EHR requirements
Jha et al [57]	Cross-sectional	COVID-19 pandemic and in-house billing	Stay positive; improved EHR design	Documentation through EHR
Esmaeilzadeh and Mirzaei [58]	Cross-sectional	Less direct communication with patients, inadequate training for using HIT, and increasing computerization at work	Positive perceptions of EHR; more policy and legal interventions to ensure meaningful use of EHR	Poor EHR usability; time spent entering data
Holzer et al [59]	Cross-sectional	Receive COVID-19 patients	Using EHR to streamline clinical care activities; physician task relief	Using EHR outside work; increased EHR workload
Wilkie et al [60]	Cross-sectional	High workload and insufficient resources	Good leadership; prioritize work-life balance	Poor EHR usability
Eschenroeder et al [61]	Case-control	Specialty	Organizational support for EHR	After-hours EHR charting time per week >6 hours; time-consuming data entry
Sharp et al [62]	Case-control	Working hours per week >70 hours	Report system to cover personal illness or emergency; access to mental health services; reduce EHR and clerical burden	>90 minutes on the EHR outside of the workday
Peccoralo et al [63]	Case-control	Clerical work time (>60 minutes/day) and poorer work-life integration	Reducing time spent on EHR and clerical tasks	Using EHR outside working (>90 minutes/day); EHR adds to daily work frustration
Harris et al [64]	Case-control	Insufficient time for documentation	Improve EHR usability; documentation practices optimization	Using EHR outside working or at home; EHR adding to daily frustration
Robertson et al [65]	Case-control	Dissatisfaction with work-life balance and female gender	EHR proficiency training	Extra time spent on EHR per week >6 hours

^a^EHR: electronic health record.

^b^NICU: neonatal intensive care unit.

^c^CPOE: computerized physician order entry.

^d^HIT: health information technology.

^e^CLOC: clinician logged-in outside clinic time.

**Table 4:** Proposed solutions for burnout mentioned.

Perspectives/solutions and suggestions		Measures
**EHR^a^**		
	Improve EHR usability and performance	Enhance EHR user interface and design to reduce health care professionals to use
	Institutions provide timely technical support during EHR use	Improving the effectiveness and efficiency of technological responses
	Institutions should offer comprehensive training courses for EHR users	Ensure users master EHR skills to reduce burnout from technological issue
**Working environment and organizational support**		
	Institutions introduce mechanisms for regular assessment of EHR efficacy	Regularly optimize and update the system based on user feedback
	Establish a schedule, routine, and workflow	Design and optimize the workflow to ensure that the EHR aligns with the health care professional’s actual work, reducing unnecessary steps and improving work efficiency
	Enhance peer, managerial, and technical support	Provide appropriate human resources, such as medical assistants, scribes, and improving teamwork to distribute workload among health care professionals
	Development of transparent policies and objectives	Establish clear policies and legislation to define the purpose, scope, and duration of EHR use, to delineate the responsibilities and obligations of health care professionals, and to reduce confusion and burnout
**Personal**		
	Use of mental health resources and services	Counseling services and mindfulness meditation therapy help health care professionals better manage work stress and reduce their psychological distress
	Encourage academic and career development	Plan career paths and training programs and create an environment for career development and learning

^a^EHR: electronic health record.

## Discussion

### Key Findings

This study explores the relationship between burnout and health care professionals. Our analysis revealed several key findings. First, the prevalence of burnout differs between assessment instruments, with the MBI-HSS indicating higher levels of burnout. However, this difference was not statistically significant. Second, there was a positive association between the average daily duration of EHR use and the risk of burnout. In particular, reducing the administrative burnout emerged as an effective strategy to reduce the risk of burnout [63]. Third, positive perceptions of the EHR and constructive work attitudes were correlated with the reduction in burnout.

The MBI-HSS is valued for its extensive validation and widespread acceptance as an essential tool for assessing burnout. Our findings suggest that the MBI-HSS may report higher rates of burnout due to several factors: sensitivity to burnout constructs—unlike self-report measures, which may rely predominantly on respondents' respondents’ subjective feelings, the MBI-HSS comprehensively assesses burnout across multiple dimensions: emotional exhaustion, depersonalization, and personal accomplishment. This multidimensional assessment provides a nuanced perception of burnout, encompassing both its physical and psychological facets. These include the following: standardized cut-off scores—the MBI-HSS delineates specific cut-off scores for its dimensions, establishing clear criteria for identifying significant levels of burnout. This standardization promotes a consistent classification framework for burnout, which may contribute to the higher prevalence rates reported. Comprehensive assessment—the multidimensional approach of the MBI-HSS allows for a comprehensive assessment of burnout, including emotional exhaustion, depersonalization, and personal accomplishment. This thorough assessment is able to uncover more precise and detailed manifestations of burnout, thereby increasing detection rates. Benchmark for comparison—the MBI-HSS is often used as a benchmark for validating alternative burnout measures, and differences in results when compared with other instruments do not necessarily indicate a variance in prevalence. Rather, these differences underscore the accuracy of the MBI-HSS and the comprehensive scope of its assessment. The use of different instruments underlines the heterogeneity observed in our study results.

### Solutions

This study demonstrates a robust relationship between workload, time spent using EHR, and burnout. Through a systematic review, we outline several pragmatic recommendations aimed at mitigating these problems.

#### Reduce Documentation and EHR Workload

A key strategy for alleviating workload concerns is to adopt a rational task allocation and effective teamwork model. Previous research highlights the effectiveness of this approach in reducing workload pressures [33,53]. By integrating medical assistants and scribes into the health care team, it is possible to distribute clerical tasks more evenly, thereby reducing the burden on health care professionals. This redistribution not only reduces workload but also increases overall operational efficiency [53,69,70]. In addition, the provision of targeted training is critical to improving teamwork dynamics, communication skills, and workflow efficiency. Such training efforts aim to cultivate a competent team capable of optimizing and streamlining workflow processes. The ultimate goal is to minimize documentation and EHR-related workloads, thereby making a significant contribution to reducing burnout among health care professionals [58,63].

#### Optimizing EHR and Training Courses

Continuous refinement of EHR systems through improved design, functionality, and integration of predesigned templates and phrases effectively increases system efficiency. The elimination of redundant steps and interactions further improves the user experience [32,71]. For example, customizing templates to include commonly used medical advice and alerts tailored to the specific needs of different departments significantly increases EHR efficiency [48,72]. Numerous studies have highlighted the critical role of improving user interaction with the EHR system. Developing a user-friendly interface that minimizes unnecessary clicks and reduces redundant and irrelevant data entry has been shown to significantly improve the user experience. Such improvements also significantly reduce the cognitive burden on health care professionals, resulting in a more streamlined and efficient health care delivery process [32,39,42]. In addition, comprehensive training and strong technical support are critical to improving the efficiency and effectiveness of EHR use. Systematic training aimed at promoting EHR proficiency among health care professionals can significantly improve operational efficiency and mitigate the effects of technology stress [46,58]. Research emphasizes the importance of training health care professionals to enhance EHR use and tailoring templates to specific clinical workflows.

### Artificial Intelligence–Based Solutions

The integration of artificial intelligence (AI) into EHR systems represents a significant frontier for improvement. Innovations in machine learning, natural language processing (NLP), and large language models (LLMs) are poised to significantly increase the intelligence and automation capabilities of EHR systems [73,74]. Incorporating speech recognition and automated dictation or note-taking into hospital workflows can streamline the creation of medical documents, thereby increasing operational efficiency [75]. NLP is characterized by its ability to efficiently organize both unstructured and semistructured textual records, thereby facilitating a reduction in paperwork [76,77]. Recent research has highlighted the utility of LLMs, such as GPT-4, as powerful tools for medical documentation [78,79]. The use of technologies such as GPT-4 as a linguistic assistant or the use of intelligent templates can significantly speed up the medical documentation process for health care professionals, while improving the accuracy of documentation [79]. In addition, the researchers developed a data-driven method to generate recommendations for refining alert criteria through an explainable AI framework [80]. This advancement directly addresses the issue of overalerting in clinical decision support systems, which has been identified as a potential contributor to burnout among health care professionals. By reducing unnecessary alerts, this approach promises to reduce the cognitive and operational workload of health care professionals, thereby improving both the quality of patient care and the work-life balance of health care staff. While AI technology could potentially help reduce burnout, it is important to recognize that the causes of burnout are complex and require further research.

### Implications for Future Research

There is considerable evidence to support the need for comprehensive redesign of EHR systems to improve efficiency [32,51,53,81]. However, the literature reveals a paucity of published empirical research quantifying EHR limitations, user fatigue and burnout. While some studies have indirectly demonstrated the poor usability of EHR by measuring pupillary reflex and cognitive fatigue [82,83], claims of inefficiency are primarily based on subjective perceptions of users. Thus, there is a need for more studies that objectively assess usability and user experience. Future research should aim to quantitatively assess the usability of EHR systems and their impact on the physical and mental well-being of health care professionals.

Furthermore, the incorporation of AI, specifically LLMs, into EHR systems is an important future research direction to reduce burnout among health care professionals. Such research could include, but is not limited to, (1) reducing the amount of time health care professionals spend on nonclinical tasks by automating administrative tasks, including data entry, scheduling, and patient history taking; (2) using LLMs to efficiently generate and review medical documentation to ensure high quality and consistency of documentation while saving time; (3) improving the interpretability and transparency of clinical decision support to provide clinicians with trustworthy decision support to reduce their cognitive load; and (4) ensuring the ethical use of AI to guarantee that AI systems are used ethically and that algorithms are unbiased. The integration of AI into EHR systems must comply with strict privacy regulations to protect patient privacy [84]. Exploring the potential of AI could make a significant contribution to creating a more supportive and efficient health care ecosystem [73,79,85].

### Limitations

This review has several limitations. First, it has a language bias by including only peer-reviewed literature published in English. This limitation may introduce information and selection bias by omitting non-English studies that may provide valuable insights or alternative viewpoints on the topic. Second, the internal heterogeneity of the included studies is remarkably high, with significant differences in methodology, participant demographics, and outcome measures between studies, which may bias the synthesis of findings. In addition, the geographical distribution of the selected studies is dominated by North American research, with only 1 study from Iran. This distribution may introduce regional bias, as health care practices and experiences in these areas may not accurately reflect global patterns.

In addition, the temporal scope of the study, covering the years 2020 to 2022, was significantly influenced by the COVID-19 pandemic. Data collected during this period may be subject to bias or inaccuracy due to the unprecedented impact of the pandemic on global health systems. Additionally, the pandemic introduced new stressors and challenges for health care professionals, which may have influenced the incidence and manifestation of their burnout. These factors should be carefully considered when interpreting the study results, as they may limit the generalizability and significance of the findings beyond the specific context and timeframe of the pandemic.

### Conclusions

This review highlights the significant impact of the EHR and the workload of health care professionals on burnout and emphasizes the need for targeted solutions such as workflow optimization, improved training, and the use of medical scribes. It also identifies that the potential of AI to improve EHR efficiency is a promising direction. Despite these findings, there remains a critical need for empirical research to accurately quantify the challenges associated with EHR use and their impact on provider well-being. Future studies are encouraged to explore innovative solutions to foster a more supportive health care environment.

## Appendix

Multimedia Appendix 1 PRISMA (Preferred Reporting Items for Systematic Reviews and Meta-Analyses) checklist.

Multimedia Appendix 2 PROSPERO registration.

Multimedia Appendix 3 Search strategy.

Multimedia Appendix 4 Joanna Briggs Institute checklist for the cross-sectional studies included.

Multimedia Appendix 5 NOS results for the case-control studies included.

Multimedia Appendix 6 Joanna Briggs Institute Prevalence Critical Appraisal Tool.

Multimedia Appendix 7 Basic characteristics of the studies included.

Multimedia Appendix 8 Funnel plot for the studies included.

Multimedia Appendix 9 The results of the publication bias test.

## Abbreviations

AI: artificial intelligence
EHR: electronic health record
EMR: electronic medical record
IV: inverse variation methods
JBI: Joanna Briggs Institute
LLM: large language model
MBI-HSS: Maslach Burnout Inventory-Human Services Survey instrument
NLP: natural language processing
NOS: Newcastle-Ottawa Scale
OR: odds ratio
PRISMA: Preferred Reporting Items for Systematic Reviews and Meta-Analysis

## Data Availability

All data generated or analyzed during this study are included in this published article and its supplementary information files.
