# Supplementary material for: Evaluating the Prevalence of Burnout Among Health Care Professionals Related to Electronic Health Record Use: Systematic Review and Meta-Analysis
Source: JMIR Med Inform. 2024 Jun 12;12:e54811. doi: 10.2196/54811 (PMC11208837; doi:10.2196/54811)
Supplement: Multimedia Appendix 2 [file medinform_v12i1e54811_app2.pdf]

Systematic review

A list of fields that can be edited in an update can be found here

1. * Review title.

Give the title of the review in English

A systematic review and meta-analysis of burnout and alert fatigue of clinicians caused by clinical decision support system

2. Original language title.

For reviews in languages other than English, give the title in the original language. This will be displayed with the English language title.

3. * Anticipated or actual start date.

Give the date the systematic review started or is expected to start.

01/09/2021

4. * Anticipated completion date.

Give the date by which the review is expected to be completed.

31/12/2021

5. * Stage of review at time of this submission.

This field uses answers to initial screening questions. It cannot be edited until after registration.

Tick the boxes to show which review tasks have been started and which have been completed.

Update this field each time any amendments are made to a published record.

The review has not yet started: No

Review stage	Started	Completed
Preliminary searches	Yes	No
Piloting of the study selection process	Yes	No
Formal screening of search results against eligibility criteria	No	No
Data extraction	No	No
Risk of bias (quality) assessment	No	No
Data analysis	No	No

Provide any other relevant information about the stage of the review here.

6. * Named contact.

The named contact is the guarantor for the accuracy of the information in the register record. This may be any member of the review team.

Jialin Liu

Email salutation (e.g. "Dr Smith" or "Joanne") for correspondence:

Dr Liu

7. * Named contact email.

Give the electronic email address of the named contact.

dljl8@163.com

8. Named contact address

Give the full institutional/organisational postal address for the named contact.

Westchina school of Medicine, Chengdu, Sichuan Province, China

9. Named contact phone number.

Give the telephone number for the named contact, including international dialling code.

+8618980601690

10. * Organisational affiliation of the review.

Full title of the organisational affiliations for this review and website address if available. This field may be

completed as 'None' if the review is not affiliated to any organisation.

Westchina school of Medicine

Organisation web address:

http://www.wchscu.cn/public/index.html

11. * Review team members and their organisational affiliations.

Give the personal details and the organisational affiliations of each member of the review team. Affiliation refers to groups or organisations to which review team members belong. **NOTE: email and country now MUST be entered for each person, unless you are amending a published record.**

Dr Jialin Liu. Westchina school of Medicine
Miss Yuxuan Wu. Westchina school of Medicine
Ind Siru Liu. Department of Biomedical Informatics, Vanderbilt University Medical Center, Nashville, TN, USA
Ind Jili Li. West China Medical School, Sichuan University, Chengdu, China
Ind Zhan Qu. West China Medical School, Sichuan University, Chengdu, China

12. * Funding sources/sponsors.

Details of the individuals, organizations, groups, companies or other legal entities who have funded or sponsored the review.

None

Grant number(s)

State the funder, grant or award number and the date of award

None

13. * Conflicts of interest.

List actual or perceived conflicts of interest (financial or academic).

None

14. Collaborators.

Give the name and affiliation of any individuals or organisations who are working on the review but who are not listed as review team members. **NOTE: email and country must be completed for each person, unless you are amending a published record.**

15. * Review question.

State the review question(s) clearly and precisely. It may be appropriate to break very broad questions down into a series of related more specific questions. Questions may be framed or refined using PI(E)COS or similar where relevant.

The study aims to systematically review the existing evidence from RCT studies that examine the mental

alert fatigue and burnout on clinicians brought by application of clinical decision support system to inform future research.

16. * Searches.

State the sources that will be searched (e.g. Medline). Give the search dates, and any restrictions (e.g. language or publication date). Do NOT enter the full search strategy (it may be provided as a link or attachment below.)

We will search articles in database including PubMed, EMBASE , OVID and Cochrane Library for papers published between 2010.1.1 and 2021.9.1. The search string will be built as follows:(clinicians) AND (clinicial decision support) AND((burnout) OR (alert fatigue)).The electronic database search will be supplemented by a manual search of the reference lists of included articles.

17. URL to search strategy.

Upload a file with your search strategy, or an example of a search strategy for a specific database, (including the keywords) in pdf or word format. In doing so you are consenting to the file being made publicly accessible. Or provide a URL or link to the strategy. Do NOT provide links to your search **results**.

The literature search of Embase, PubMed, OVID, the Cochrane Library and CNKI will be performed individually in each database using a unique set of relevant subject titles and appropriate MeSH terms.

Alternatively, upload your search strategy to CRD in pdf format. Please note that by doing so you are consenting to the file being made publicly accessible.

Do not make this file publicly available until the review is complete

18. * Condition or domain being studied.

Give a short description of the disease, condition or healthcare domain being studied in your systematic review.

The clinical decision support system is a tool which uses clinical knowledge and health information to assist medical decision-making, thereby improving medical care services.The early application of CDS was mainly in the form of electronic medical records. With the rapid development of information technology, CDS has gradually developed to help users (patients and medical practitioners) perform intelligent health management, disease diagnosis and provide treatment decisions.Nowadays CDS is mainly used in medical workstations, allowing clinicians to combine professional knowledge with information or advice provided by CDS.Some previous studies have shown that the CDS can reduce hospitalization and outpatient costs.The above are all the benefits that CDS brings to patients. It is generally believed that CDS can help clinicians make more precise diagnosis and treatment and reduce workload.

19. * Participants/population.

Specify the participants or populations being studied in the review. The preferred format includes details of both inclusion and exclusion criteria.

Clinical medical staff

20. * Intervention(s), exposure(s).

Give full and clear descriptions or definitions of the interventions or the exposures to be reviewed. The preferred format includes details of both inclusion and exclusion criteria.

usage of clinical decision support system

21. * Comparator(s)/control.

Where relevant, give details of the alternatives against which the intervention/exposure will be compared (e.g. another intervention or a non-exposed control group). The preferred format includes details of both inclusion and exclusion criteria.

medical staff who do not use CDS in their working environment or who still provide services in accordance

with the traditional medical care model

22. * Types of study to be included.

Give details of the study designs (e.g. RCT) that are eligible for inclusion in the review. The preferred format includes both inclusion and exclusion criteria. If there are no restrictions on the types of study, this should be stated.

Randomized controlled trials (RCTs) will be included

23. Context.

Give summary details of the setting or other relevant characteristics, which help define the inclusion or exclusion criteria.

24. * Main outcome(s).

Give the pre-specified main (most important) outcomes of the review, including details of how the outcome is defined and measured and when these measurement are made, if these are part of the review inclusion criteria.

job burnout and alert fatigue of clinicians

Measures of effect

Please specify the effect measure(s) for you main outcome(s) e.g. relative risks, odds ratios, risk difference, and/or 'number needed to treat.

OR value and incidence

25. * Additional outcome(s).

List the pre-specified additional outcomes of the review, with a similar level of detail to that required for main outcomes. Where there are no additional outcomes please state ‘None’ or ‘Not applicable’ as appropriate to the review

Career satisfaction and happiness

Measures of effect

Please specify the effect measure(s) for you additional outcome(s) e.g. relative risks, odds ratios, risk difference, and/or 'number needed to treat.

26. * Data extraction (selection and coding).

Describe how studies will be selected for inclusion. State what data will be extracted or obtained. State how this will be done and recorded.

Two authors will independently extract data. Any disagreement will be resolved by discussion until consensus is reached or by consulting a third author.The following data will be extracted: author, publication date, country where the study was conducted, study design, basic information of the sample population , the control and the intervention.

27. * Risk of bias (quality) assessment.

State which characteristics of the studies will be assessed and/or any formal risk of bias/quality assessment tools that will be used.

Two reviewers will independently assess risk of bias based on the following domains from recommendations from the Cochrane handbook.We will use funnel plot and Egger’s test the evaluate the publication bias.

28. * Strategy for data synthesis.

Describe the methods you plan to use to synthesise data. This **must not be generic text** but should be **specific to your review** and describe how the proposed approach will be applied to your data. If meta-analysis is planned, describe the models to be used, methods to explore statistical heterogeneity, and software package to be used.

Odds ratio (OR) and its 95% confidence intervals (95% CI) will be calculated for the outcome in each included trial. Due to expected heterogeneity among the trials, Meta-analysis using the random-effects model will be conducted with stata 16.

29. * Analysis of subgroups or subsets.

State any planned investigation of ‘subgroups’. Be clear and specific about which type of study or participant will be included in each group or covariate investigated. State the planned analytic approach.

Three subgroup analyses will also be undertaken: The first to assess if the different number of patients produce different effects; The second to investigate whether the application of CDS in different levels of medical institutions leads to difference in incidence of alert fatigue;The third is to investigate that the influence of different types of CDS on the incidence of job burnout.

30. * Type and method of review.

Select the type of review, review method and health area from the lists below.

Type of review

Cost effectiveness

No

Diagnostic

No

Epidemiologic

No

Individual patient data (IPD) meta-analysis

No

Intervention

Yes

Living systematic review

No

Meta-analysis

Yes

Methodology

No

Narrative synthesis

No

Network meta-analysis

No

Pre-clinical

No

Prevention

No

Prognostic

No

Prospective meta-analysis (PMA)

No

Review of reviews

No

Service delivery

No

Synthesis of qualitative studies

No

Systematic review

Yes

Other

No

Health area of the review

Alcohol/substance misuse/abuse

No

Blood and immune system

No

Cancer

No

Cardiovascular

No

Care of the elderly

No

Child health

No

Complementary therapies

No

COVID-19

No

Crime and justice

No

Dental

No

Digestive system

No

Ear, nose and throat

No

Education

No

Endocrine and metabolic disorders

No

Eye disorders

No

General interest

No

Genetics

No

Health inequalities/health equity

No

Infections and infestations

No

International development

Yes

Mental health and behavioural conditions

No

Musculoskeletal

No

Neurological

No

Nursing

No

Obstetrics and gynaecology

No

Oral health

No

Palliative care

No

Perioperative care

No

Physiotherapy

No

Pregnancy and childbirth

No

Public health (including social determinants of health)

No

Rehabilitation

No

Respiratory disorders

No

Service delivery

Yes

Skin disorders

No

Social care

Yes

Surgery

No

Tropical Medicine

No

Urological

No

Wounds, injuries and accidents

No

Violence and abuse

No

31. Language.

Select each language individually to add it to the list below, use the bin icon to remove any added in error.

English

There is not an English language summary

32. * Country.

Select the country in which the review is being carried out. For multi-national collaborations select all the countries involved.

China

33. Other registration details.

Name any other organisation where the systematic review title or protocol is registered (e.g. Campbell, or The Joanna Briggs Institute) together with any unique identification number assigned by them. If extracted data will be stored and made available through a repository such as the Systematic Review Data Repository (SRDR), details and a link should be included here. If none, leave blank.

34. Reference and/or URL for published protocol.

If the protocol for this review is published provide details (authors, title and journal details, preferably in Vancouver format)

Add web link to the published protocol.

Or, upload your published protocol here in pdf format. Note that the upload will be publicly accessible.

No I do not make this file publicly available until the review is complete

Please note that the information required in the PROSPERO registration form must be completed in full even if access to a protocol is given.

35. Dissemination plans.

Do you intend to publish the review on completion?

No

Give brief details of plans for communicating review findings.?

36. Keywords.

Give words or phrases that best describe the review. Separate keywords with a semicolon or new line. Keywords help PROSPERO users find your review (keywords do not appear in the public record but are included in searches). Be as specific and precise as possible. Avoid acronyms and abbreviations unless these are in wide use.

Clinical decision support;clinician job burnout;alert fatigue;EMR

37. Details of any existing review of the same topic by the same authors.

If you are registering an update of an existing review give details of the earlier versions and include a full bibliographic reference, if available.

38. * Current review status.

Update review status when the review is completed and when it is published.New registrations must be ongoing so this field is not editable for initial submission.

Please provide anticipated publication date

Review_Ongoing

39. Any additional information.

Provide any other information relevant to the registration of this review.

40. Details of final report/publication(s) or preprints if available.

Leave empty until publication details are available OR you have a link to a preprint (NOTE: this field is not editable for initial submission). List authors, title and journal details preferably in Vancouver format.

Give the link to the published review or preprint.
