# Supplementary material for: Evaluating the Prevalence of Burnout Among Health Care Professionals Related to Electronic Health Record Use: Systematic Review and Meta-Analysis
Source: JMIR Med Inform. 2024 Jun 12;12:e54811. doi: 10.2196/54811 (PMC11208837; doi:10.2196/54811)
Supplement: Multimedia Appendix 3 [file medinform_v12i1e54811_app3.docx]

Search strategy for Pubmed on June 30, 2023

Records identified: 335 filter: published between 2009/1/1-2022/12/31 and in English

((physicians[Title/Abstract]) OR (doctors[Title/Abstract]) OR (medical staff[Title/Abstract]) OR (healthcare professional[Title/Abstract]) OR (clinicians[Title/Abstract]) OR (nurses[Title/Abstract])) AND ((electronic health record[Title/Abstract]) OR (EHR[Title/Abstract]) OR (electronic medical record[Title/Abstract]) OR (EMR[Title/Abstract]) OR (computerized physician order entry[Title/Abstract]) OR (CPOE[Title/Abstract]) OR (clinical decision support system[Title/Abstract]) OR (CDSS[Title/Abstract])) AND ((burnout[Title/Abstract]) OR (burn-out[Title/Abstract]) OR (alert fatigue[Title/Abstract]) OR (alarm fatigue[Title/Abstract]) OR (exhaustion[Title/Abstract]))

Search strategy for Embase on 30 June 2023

Records identified: 1490 filter: published between 2009/1/1-2022/12/31 and in English

(Burnout.mp. Or exp burnout/ Rr Burn-out.mp. Or Burn out.mp. Or Exhaustion.mp. Or alert fatigue.mp. Or alarm fatigue.mp.) AND (physicians.mp. Or doctors.mp. Or nurses.mp. Or clinicians.mp. Or medical staff.mp. Or healthcare professional.mp.) AND (electronic health record.mp. Or EHR.mp. Or electronic medical record.mp. Or EMR.mp. Or CPOE.mp. Or computerized physician order entry.mp Or CDSS.mp. Or clinical decision support system.mp.)

Search strategy for Web of Science on 30 June 2023

Records identified: 946 filter: published between 2009/1/1-2022/12/31 and in English

(TS=(physician) OR TS=(doctor) OR TS=(clinician) OR TS=(nurses) OR TS=(medical staff) OR TS=(healthcare professional)) AND (TS=(electronic health record) OR TS=(EHR) OR TS=(electronic medical record) OR TS=(EMR) OR TS=(CPOE) OR TS=(computerized physician order entry) OR TS=(CDSS) OR TS=(clinical decision support system)) AND (TS=(burnout) OR TS=(burn-out) OR TS=(burn out) OR TS=(exhaustion) OR TS=(alert fatigue) OR TS=(alarm fatigue))
