# Supplementary material for: Evaluating the Prevalence of Burnout Among Health Care Professionals Related to Electronic Health Record Use: Systematic Review and Meta-Analysis
Source: JMIR Med Inform. 2024 Jun 12;12:e54811. doi: 10.2196/54811 (PMC11208837; doi:10.2196/54811)
Supplement: Multimedia Appendix 4 [file medinform_v12i1e54811_app4.pdf]

No.	Title	Author	1	2	3	4	5	6	7
1	Relationship Between Clerical Burden and Characteristics of the Electronic Environment With Physician Burnout and Professional Satisfaction	Tait D Shanafelt	No	Moderate	Moderate	Moderate	Yes	Yes	Yes
2	Factors associated with provider burnout in the NICU	Daniel S Tawfik	No	No	Yes	No	Yes	Moderate	Moderate
3	Physician Burnout in Wisconsin: An Alarming Trend Affecting Physician	Anne Hauer	Moderate	Moderate	Moderate	No	Yes	Yes	Moderate
4	Association of Electronic Health Record Design and Use Factors With Clinician Stress and Burnout	Philip J Kroth	Moderate	Moderate	Moderate	Moderate	Yes	Yes	Yes
5	Burnout and EHR use among academic primary care physicians with varied clinical workloads	Brian Tran	No	Moderate	Yes	Moderate	Yes	Moderate	Yes
6	Electronic health record associated stress: A survey study of adult congenital heart disease specialists	Darcy N Marckini	Moderate	No	Moderate	Moderate	Yes	Yes	Yes
7	Cross-sectional survey of workplace stressors associated with physician burnout measured by the Mini-Z and the Maslach Burnout Inventory	Kristine Olson	Yes	Moderate	Yes	No	Yes	Yes	Moderate
8	Physician stress and burnout: the impact of health information technology	Rebekah L Gardner	Moderate	No	Yes	Moderate	Yes	Yes	Moderate
9	Physicians' Well-Being Linked To In-Basket Messages Generated By Algorithms In Electronic Health Records	Ming Tai-Seale	Yes	Moderate	Moderate	Moderate	Yes	Moderate	Moderate
10	The Influence of Electronic Health Record Use on Physician Burnout: Cross-Sectional	Tania Tajirian	No	No	Yes	No	Yes	Moderate	Moderate
11	Are specific elements of electronic health record use associated with clinician burnout more than others?	Ross W Hilliard	Moderate	No	Yes	Moderate	Moderate	Yes	Yes
12	Association Between Difficulty with VA Patient-Centered Medical Home Model Components and Provider Emotional Exhaustion and Intent to Remain in Practice	Eric A Apaydin	No	No	Yes	Yes	Yes	Moderate	Moderate
13	Frequency and Causes of Burnout in US Community Oncologists in the Era of Electronic Health Records	Ajeet Gajra	No	No	Moderate	No	Yes	Moderate	Yes
14	Electronic health records and burnout: Time spent on the electronic health record after hours and message volume associated with exhaustion but not with cynicism among primary care clinicians	Julia Adler-Milstein	Moderate	No	Moderate	Yes	Moderate	Yes	Yes
15	Use of Health Information Technology by Rhode Island Physicians and Advanced Practice Providers, 2019	Brittany Mandeville	Moderate	Moderate	Yes	No	Yes	No	Moderate
16	The Effect of COVID-19 on Interventional Pain Management Practices: A Physician Burnout Survey	Sachin Sunny Jha	No	No	No	No	Yes	No	Yes
17	High Burden of Burnout on Rheumatology Practitioners	Vivekanand Tiwari	No	No	Yes	No	Moderate	Moderate	Yes
18	Burnout Among United States Orthopaedic Surgery Residents	Jeremy S Somerson	Moderate	Yes	Yes	No	Yes	Moderate	Yes
19	The Association Between Perceived Electronic Health Record Usability and Professional Burnout Among US Physicians	Edward R Melnick	Yes	Moderate	Yes	Moderate	Yes	Moderate	Yes
20	Using Electronic Health Records to Mitigate Workplace Burnout Among Clinicians During the COVID-19	Pouyan Esmaeilzadeh	Moderate	Moderate	Yes	Moderate	Yes	Yes	Yes
21	Vascular surgeon wellness and burnout: A report from the Society for Vascular Surgery Wellness Task Force	Dawn M Coleman	Moderate	No	Yes	Moderate	Yes	Moderate	Yes
22	Novel Nonproprietary Measures of Ambulatory Electronic Health Record Use Associated with Physician Work Exhaustion	Amrita Sinha	No	Moderate	Yes	No	Yes	Moderate	Moderate
23	Impact of Changes in EHR Use during COVID-19 on Physician Trainee Mental Health	Katherine J. Holzer	Moderate	No	Yes	No	Yes	Yes	Moderate
24	Use of multifunctional electronic health records and burnout among primary care nurse practitioners	Cilgy M Abraham	Moderate	No	Yes	Moderate	Moderate	Moderate	Yes
25	Frustration With Technology and its Relation to Emotional Exhaustion Among Health Care Workers: Cross-sectional	Daniel S Tawfik	Moderate	Yes	Yes	Moderate	Moderate	Yes	Yes
26	Evolution of a physician wellness, engagement and excellence strategy: lessons learnt in a mental health setting	Treena Wilkie	Yes	No	Yes	No	Yes	Yes	Moderate
27	Burnout Among US Gastroenterologists and Fellows in Training: Identifying Contributing Factors and Offering Solutions	Joseph C Anderson	Moderate	Moderate	No	No	Yes	Moderate	Yes
28	Perceived Value of the Electronic Health Record and Its Association with Physician Burnout	Maria Livaudais	No	Moderate	Yes	Moderate	Yes	Yes	Yes
29	Burnout, Professional Fulfillment, Intention to Leave, and Sleep-Related Impairment among Radiology Trainees across the United States (US): A Multisite	Mikhail C S S Higgins	Moderate	No	Yes	Moderate	Yes	Yes	Yes
30	Burnout in Pediatric Emergency Medicine Physicians: A Predictive Model	Janienne E Kondrich	Yes	Moderate	Yes	No	Yes	Yes	Yes
31	Hospitalist Perceptions of Electronic Health Records: a Multi-site Survey	Zuzanna Czernik	No	Moderate	No	No	Yes	Moderate	Moderate
32	Burnout Among Nephrologists in the United States: A Survey Study	Devika Nair	Moderate	Yes	Yes	Moderate	Yes	Yes	Yes
