# Supplementary material for: Evaluating the Prevalence of Burnout Among Health Care Professionals Related to Electronic Health Record Use: Systematic Review and Meta-Analysis
Source: JMIR Med Inform. 2024 Jun 12;12:e54811. doi: 10.2196/54811 (PMC11208837; doi:10.2196/54811)
Supplement: Multimedia Appendix 5 [file medinform_v12i1e54811_app5.pdf]

No.	Title	First author	year	Region	Sample.size	Participants	Exposure	burnout prevalence	OR	LCI	UCI	Is the case definition adequate?	Representativeness of the cases	Selection of control	Definition of controls	Comparability of cases and controls on the basis of design or analysis	Ascertainment of exposure	Same method of ascertainment for cases and controls	Non-response rate	Total
33	Associations of physician burnout with organizational electronic health record support and after-hours charting	H.C Eschenroeder	2020	America	25018	physicians	after-hours EHR charting time per week>6h	30.44%	2.43	2.30	2.57	1	1	1	0	2	1	1	0	7
34	A National Survey of Burnout and Depression Among Fellows Training in Pulmonary and Critical Care Medicine: A Special Report by the Association of Pulmonary and Critical Care Medicine Program Directors	Michelle Sharp	2019	America	502	medic fellow	working hours per week>70h	31.67%	2.80	1.78	4.40	1	0	0	1	1	1	1	0	5
35	The impact of time spent on the electronic health record after work and of clerical work on burnout among clinical	Lauren A Peccoralo	2019	America	1346	clinical faculty	time spent on EHR outside work>90min	28.60%	1.90	1.40	2.78	1	1	1	0	1	1	1	0	6
36	Estimating the association between burnout and electronic health record-related stress among advanced practice	Daniel A.Harris	2017	America	333	advanced practice registered nurses	insufficient time for EHR documentation	20.72%	3.72	1.78	7.80	1	1	0	1	1	0	1	0	5
37	Electronic Health Record Effects on Work-LifeBalance and Burnout Within the I3 Population Collaborative	Sandy L.Robertson	2015	America	585	primary care residents and faculty	extra time spent on EHR per week>6h	36.92%	2.90	1.90	4.40	1	1	1	0	1	1	1	0	6
