# Supplementary material for: Evaluating the Prevalence of Burnout Among Health Care Professionals Related to Electronic Health Record Use: Systematic Review and Meta-Analysis
Source: JMIR Med Inform. 2024 Jun 12;12:e54811. doi: 10.2196/54811 (PMC11208837; doi:10.2196/54811)
Supplement: Multimedia Appendix 6 [file medinform_v12i1e54811_app6.docx]

Joanna Briggs Institute Prevalence Critical Appraisal Tool

**1. Were study participants selected in an appropriate way?**

‘Yes’ criteria (all criteria must be met):

- The source of participants is representative.
- Complete sample or random sample with a detailed instruction of sampling technique.

‘No’ criteria (at least one criteria must be met):

- The source of participants is not specified and cannot be obtained from original authors.
- Non-random sample.

Moderate criteria:

- The conditions of study are somewhere in between the above two criteria.

**2. Were the inclusion and exclusion criteria described in detail clearly?**

‘Yes’ criteria (all criteria must be met):

- The inclusion and exclusion criteria of participants are explained clearly.

‘Moderate’ criteria:

- Only one criteria is mentioned.
- The inclusion and exclusion criteria of participants was described roughly, not clearly.

‘No’ criteria:

- At least one of indicated above of participants or setting is not reported.

**3. Was the condition measured in a valid, reliable way?**

‘Yes’ criteria (all criteria must be met):

- The outcome assessment procedure was the same for all participants.
- The measurement tools were proved to be reliable and valid.

‘No’ criteria (at least one criteria must be met):

- The outcome assessment procedure was inconsistent.
- The measurement tools were not tested in previous studies.

**Note:** It is hardly possible to list the burnout assessment procedure differences comprehensively. For example, questionnaire forms (electronic vs paper-and-pencil), circumstances (interviewer’s office vs home) or deadlines (a week vs few hours) may differ. We will explain what exactly the differences are, in case of revealing.

As far as we know, most of the burnout measurement instruments are self-reported questionnaires. Therefore, if any included studies will use an instrument that requires an interview to be completed, we will invert the second ‘Yes’/’No’ criteria for this study.

**4.Was the statistical analysis and methods reported adequately?**

‘Yes’ criteria (all criteria must be met):

- The software used is reported.
- Continuous data presented in the form of mean value/median and standard deviation/ interquartile range or range.
- At least absolute rates are reported or calculable for categorical data.
- Results of burnout measurement correctly presented in at least one of these two forms.

‘No’ criteria (at least one criteria must be met):

- The software used is not reported.
- Descriptive statistics for characteristics of studies and the results of burnout measurement are not presented or not presented completely or clearly either.

**5. Were the response rate and comparability between respondents and non-respondents reported?**

‘Yes’ criteria (all criteria must be met):

- The response rate is reported and discussed in terms of reasons of its level and its impact on conclusions.
- The responders and non-responders were compared at least by socio-demographic characteristics.

‘No’ criteria (at least one criteria must be met):

- Authors did not discuss the level of response rate and its impact on conclusions.
- The responders and non-responders were not compared.

‘Moderate’ criteria:

- Response rates were mentioned but responders and non-responders were not compared.

1. **Whether to report the ethical review status？**

‘Yes’ criteria (all criteria must be met):

- The ethical review was reported in full form.
- Review material or instructions for access are provided in the attachment or main text.

‘No’ criteria:

- The ethical information was completely unmentioned.

‘Moderate’ criteria:

- It is noted that the study met the ethical review but no detailed materials or access obtained.

1. **Were the findings or results presented properly?**

‘Yes’ criteria:

- The results were presented in charts or tables with appropriate texts explained.
- The inferences and results were reported separately.

‘No’ criteria(at least one criteria must be met):

- The results were not presented completely.

‘Moderate’ criteria:

- The inferences and results were indicated but not reported separately.
