# Supplementary material for: Evaluating the Prevalence of Burnout Among Health Care Professionals Related to Electronic Health Record Use: Systematic Review and Meta-Analysis
Source: JMIR Med Inform. 2024 Jun 12;12:e54811. doi: 10.2196/54811 (PMC11208837; doi:10.2196/54811)
Supplement: Multimedia Appendix 7 [file medinform_v12i1e54811_app7.pdf]

No.	Title	First Author	Year of data collection	Study design	Region	Sample size	cases	prevalence	Response rate	Measurement	Participants	definition
1	Relationship Between Clerical Burden and Characteristics of the Electronic Environment With Physician Burnout and Professional	Tait D Shanafelt	2014	cross-sectional study	US	6560	3586	54.66%	19.20%	MBI	physicians	The scores meet either definition of high EE or high DP
2	Factors associated with provider burnout in the NICU	Daniel S Tawfik	2011	cross-sectional study	US	1934	517	26.73%	70.00%	MBI	physicians snd other clinician staff	The scores meet the definition of high EE
3	Physician Burnout in Wisconsin: An Alarming Trend Affecting Physician Wellness	Anne Hauer	2018	cross-sectional study	US	1165	624	53.56%	8.86%	mini-Z	physicians	The burnout-related questions on the mini-Z scale scored ≥2
4	Association of Electronic Health Record Design and Use Factors With Clinician Stress and Burnout	Philip J Kroth	2019	cross-sectional study	US	282	127	45.04%	44.10%	Other	physicians snd other clinician staff	Meet the definitions of burnout on the other scales
5	Burnout and EHR use among academic primary care physicians with varied clinical workloads	Brian Tran	2017	cross-sectional study	US	107	41	38.32%	56.00%	mini-Z	physicians snd other clinician staff	The burnout-related questions on the mini-Z scale scored ≥2
6	Electronic health record associated stress: A survey study of adult congenital heart disease specialists	Darcy N Marckini	2017	cross-sectional study	Canada and US	110	44	40.00%	28.70%	MBI	physicians	The scores meet either definition of high EE or high DP
7	Cross-sectional survey of workplace stressors associated with physician burnout measured by the Mini-Z and the Maslach Burnout Inventory	Kristine Olson	2016	cross-sectional study	US	557	267	47.94%	44.00%	MBI	physicians	The scores meet either definition of high EE or high DP
8	Physician stress and burnout: the impact of health information technology	Rebekah L Gardner	2017	cross-sectional study	US	1792	465	25.95%	42.70%	mini-Z	physicians	The burnout-related questions on the mini-Z scale scored ≥2
9	Physicians' Well-Being Linked To In-Basket Messages Generated By Algorithms In Electronic Health Records	Ming Tai-Seale	2016	cross-sectional study	US	919	331	36.02%	71.13%	mini-Z	physicians	The burnout-related questions on the mini-Z scale scored ≥2
10	The Influence of Electronic Health Record Use on Physician Burnout: Cross-Sectional Survey	Tania Tajirian	2019	cross-sectional study	Canada	208	51	24.52%	43.80%	mini-Z	physicians and trainee	The burnout-related questions on the mini-Z scale scored ≥2
11	Are specific elements of electronic health record use associated with clinician burnout more than others?	Ross W Hilliard	2017	cross-sectional study	US	422	116	27.49%	39.30%	mini-Z	physicians snd other clinician staff	The burnout-related questions on the mini-Z scale scored ≥2
12	Association Between Difficulty with VA Patient-Centered Medical Home Model Components and Provider Emotional Exhaustion and Intent to Remain in Practice	Eric A Apaydin	2016	cross-sectional study	US	116	62	53.45%	Not reported	MBI	physicians snd other clinician staff	The scores meet the definition of high EE
13	Frequency and Causes of Burnout in US Community Oncologists in the Era of Electronic	Ajeet Gajra	2018	cross-sectional study	US	163	81	49.69%	Not reported	Other	physicians	self-reported burnout
14	Electronic health records and burnout: Time spent on the electronic health record after hours and message volume associated with exhaustion but not with cynicism among primary care clinicians	Julia Adler-Milstein	2018	cross-sectional study	US	122	44	36.07%	37.00%	MBI	physicians	The scores meet the definition of high EE
15	Use of Health Information Technology by Rhode Island Physicians and Advanced Practice Providers, 2019	Brittany Mandeville	2019	cross-sectional study	US	2468	539	21.84%	39.50%	mini-Z	physicians snd other clinician staff	The burnout-related questions on the mini-Z scale scored ≥2
16	The Effect of COVID-19 on Interventional Pain Management Practices: A Physician Burnout	Sachin Sunny Jha	2020	cross-sectional study	US	100	52	52.00%	55.90%	Other	physicians snd other clinician staff	self-reported burnout
17	High Burden of Burnout on Rheumatology Practitioners	Vivekanand Tiwari	2019	cross-sectional study	US	128	65	50.78%	56.00%	MBI	physicians snd other clinician staff	The scores meet any definition of high EE, high DP, or low PA
18	Burnout Among United States Orthopaedic Surgery Residents	Jeremy S Somerson	2018	cross-sectional study	US	203	78	38.42%	Not reported	MBI	residents	The scores meet either definition of high EE or high DP
19	The Association Between Perceived Electronic Health Record Usability and Professional Burnout Among US Physicians	Edward R Melnick	2018	cross-sectional study	US	870	397	45.63%	69.60%	MBI	physicians	The scores meet either definition of high EE or high DP
20	Using Electronic Health Records to Mitigate Workplace Burnout Among Clinicians During the COVID-19 Pandemic: Field Study in Iran	Pouyan Esmaeilzadeh	2020	cross-sectional study	Iran	368	134	36.41%	Not reported	Other	physicians snd other clinician staff	self-reported burnout

21	Vascular surgeon wellness and burnout: A report from the Society for Vascular Surgery Wellness Task Force	Dawn M Coleman	2018	cross-sectional study	US	872	360	41.28%	34.30%	MBI	physicians	The scores meet either definition of high EE or high DP
22	Novel Nonproprietary Measures of Ambulatory Electronic Health Record Use Associated with Physician Work Exhaustion	Amrita Sinha	2019	cross-sectional study	US	856	276	32.24%	73.00%	Other	physicians	Meet the definitions of burnout on the other scales
23	Impact of Changes in EHR Use during COVID-19 on Physician Trainee Mental Health	Katherine J. Holzer	2020	cross-sectional study	US	222	84	37.84%	16.20%	Other	physicians and trainee	Meet the definitions of burnout on the other scales
24	Use of multifunctional electronic health records and burnout among primary care nurse practitioners	Cilgy M Abraham	2018	cross-sectional study	US	396	100	25.25%	Not reported	mini-Z	nurses	The burnout-related questions on the mini-Z scale scored ≥2
25	Frustration With Technology and its Relation to Emotional Exhaustion Among Health Care Workers: Cross-sectional Observational Study	Daniel S Tawfik	2015	cross-sectional study	US	15505	5065	32.67%	70.40%	MBI	physicians snd other clinician staff	The scores meet the definition of high EE
26	Evolution of a physician wellness, engagement and excellence strategy: lessons learnt in a mental health setting	Treena Wilkie	2021	cross-sectional study	Canada	103	41	39.81%	40.90%	MBI	physicians	The scores meet either definition of high EE or high DP
27	Burnout Among US Gastroenterologists and Fellows in Training: Identifying Contributing Factors and Offering Solutions	Joseph C Anderson	2019	cross-sectional study	US	756	373	49.34%	9.20%	MBI	physicians and trainee	The scores meet either definition of high EE or high DP
28	Perceived Value of the Electronic Health Record and Its Association with Physician Burnout	Maria Livaudais	2016	cross-sectional study	US	281	127	45.20%	44.00%	Other	physicians snd other clinician staff	Meet the definitions of burnout on the other scales
29	Burnout, Professional Fulfillment, Intention to Leave, and Sleep-Related Impairment among Radiology Trainees across the United States (US): A Multisite Epidemiologic Study	Mikhail C S S Higgins	2017	cross-sectional study	US	230	86	37.39%	Not reported	Other	residents	Meet the definitions of burnout on the other scales
30	Burnout in Pediatric Emergency Medicine Physicians: A Predictive Model	Janienne E Kondrich	2018	cross-sectional study	Canada and US	416	206	49.52%	59.40%	MBI	physicians	The scores meet any definition of high EE, high DP, or low PA
31	Hospitalist Perceptions of Electronic Health Records: a Multi-site Survey	Zuzanna Czernik	2017	cross-sectional study	US	84	30	35.71%	67.00%	Other	residents	self-reported burnout
32	Burnout Among Nephrologists in the United States: A Survey Study	Devika Nair	2019	cross-sectional study	US	457	106	23.19%	Not reported	MBI	physicians	The scores meet either definition of high EE or high DP
33	Associations of physician burnout with organizational electronic health record support and after-hours charting	H.C Eschenroeder	2020	case-control study	US	25018	7616	30.44%	54.39%	mini-Z	physicians	The burnout-related questions on the mini-Z scale scored ≥2
34	A National Survey of Burnout and Depression Among Fellows Training in Pulmonary and Critical Care Medicine: A Special Report by the Association of Pulmonary and Critical Care Medicine Program Directors	Michelle Sharp	2019	case-control study	US	502	159	31.67%	51.00%	MBI	physicians and trainee	The scores meet any definition of high EE, high DP, or low PA
35	The impact of time spent on the electronic health record after work and of clerical work on burnout among clinical faculty	Lauren A Peccoralo	2019	case-control study	US	1346	385	28.60%	42.90%	MBI	physicians snd other clinician staff	The scores meet any definition of high EE, high DP, or low PA
36	Estimating the association between burnout and electronic health record-related stress among advanced practice registered nurses	Daniel A.Harris	2017	case-control study	US	333	69	20.72%	31.00%	mini-Z	nurses	The burnout-related questions on the mini-Z scale scored ≥2
37	Electronic Health Record Effects on Work-LifeBalance and Burnout Within the I3 Population Collaborative	Sandy L.Robertson	2015	case-control study	US	585	214	36.58%	68.00%	Other	physicians	Meet the definitions of burnout on the other scales
