# Supplementary figures and images for: Evaluating the Prevalence of Burnout Among Health Care Professionals Related to Electronic Health Record Use: Systematic Review and Meta-Analysis
Source: JMIR Med Inform. 2024 Jun 12;12:e54811. doi: 10.2196/54811 (PMC11208837; doi:10.2196/54811)

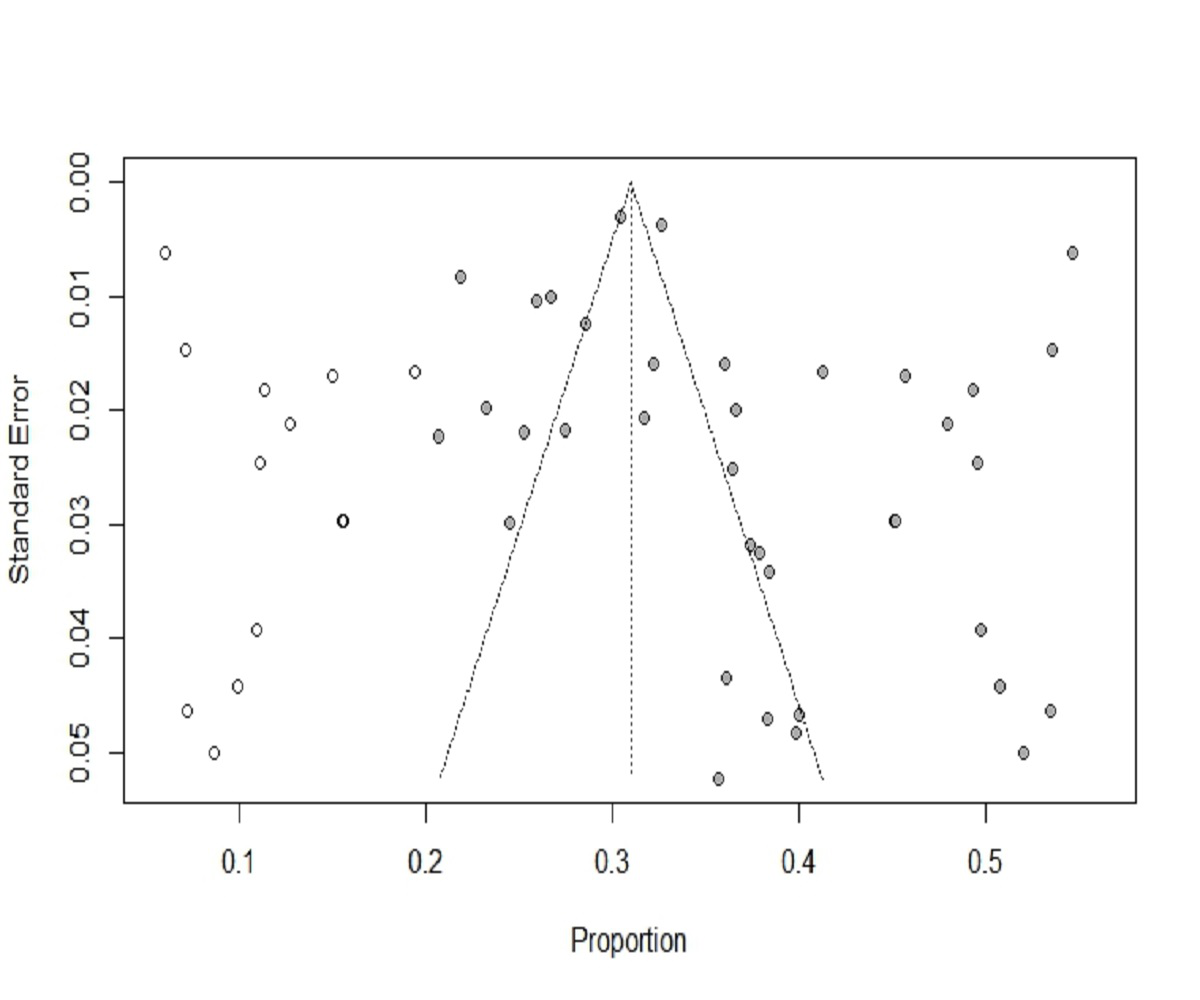

Supplement: Multimedia Appendix 8 [file medinform_v12i1e54811_app8.png]

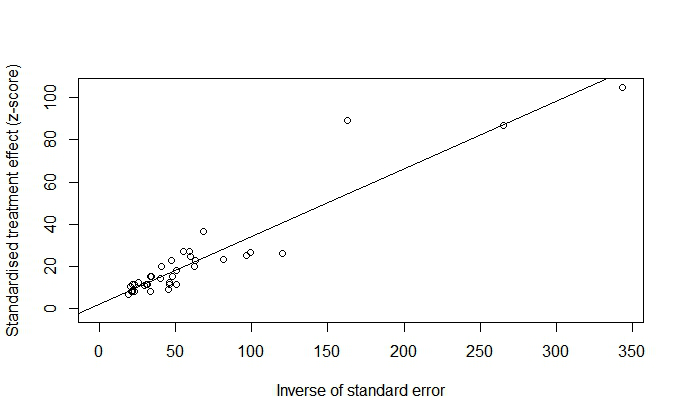

Supplement: Multimedia Appendix 9 [file medinform_v12i1e54811_app9.png]
